# An exact algorithm to find a maximum weight clique in a weighted undirected graph

**DOI:** 10.1038/s41598-024-59689-x

**Published:** 2024-04-20

**Authors:** Kati Rozman, An Ghysels, Dušanka Janežič, Janez Konc

**Affiliations:** 1 Faculty of Mathematics, Natural Sciences and Information Technologies, University of Primorska, Glagoljaška Ulica 8, 6000 Koper, Slovenia; 2 IBiTech – BioMMedA Group, Ghent University, Corneel Heymanslaan 10, Entrance 36, 9000 Gent, Belgium; 3 Theory Department, National Institute of Chemistry, Hajdrihova 19, 1000 Ljubljana, Slovenia; 4 Faculty of Pharmacy, University of Ljubljana, Aškerčeva 7, 1000 Ljubljana, Slovenia

**Keywords:** Applied mathematics, Computational science, Computer science

## Abstract

We introduce a new algorithm MaxCliqueWeight for identifying a maximum weight clique in a weighted graph, and its variant MaxCliqueDynWeight with dynamically varying bounds. This algorithm uses an efficient branch-and-bound approach with a new weighted graph coloring algorithm that efficiently determines upper weight bounds for a maximum weighted clique in a graph. We evaluate our algorithm on random weighted graphs with node counts up to 10,000 and on standard DIMACS benchmark graphs used in a variety of research areas. Our findings reveal a remarkable improvement in computational speed when compared to existing algorithms, particularly evident in the case of high-density random graphs and DIMACS graphs, where our newly developed algorithm outperforms existing alternatives by several orders of magnitude. The newly developed algorithm and its variant are freely available to the broader research community at http://insilab.org/maxcliqueweight, paving the way for transformative applications in various research areas, including drug discovery.

## Introduction

The Maximum Weight Clique Problem (MWCP) finds significant utility in bioinformatics and drug design playing a crucial role in the analysis of complex molecular networks and the identification of functionally relevant components within them. In the context of biological networks such as protein-protein interaction networks or gene regulatory networks, the MWCP is employed to uncover groups of molecules that form cohesive functional units, often associated with particular cellular processes or pathways^1^. By assigning weights to these molecules based on attributes such as gene expression levels, interaction strengths, or molecular properties, the MWCP aids in pinpointing the most influential or interconnected subsets of molecules. This knowledge is instrumental in understanding intricate biological mechanisms, disease pathways, and drug target identification.

Furthermore, the MWCP assists in the rational design of drugs by helping researchers identify clusters of interacting molecules that could be potential drug targets or that contribute to disease progression^2,3^. Thus, the application of the MWCP in bioinformatics and drug design enhances our ability to decipher complex biological systems and facilitates the development of innovative therapeutic strategies. The MWCP is also applied in other research areas, such as robotics^4^, combinatorial auctions^5^, telecommunications^6^ and protein functional sites recognition^7^.

In a weighted graph, vertices are assigned numerical weights, often positive integer values, indicating their importance, value, or cost. A maximum weight clique is a subset of vertices that meets the definition of a clique, that is, each vertex is directly connected to every other vertex in the subset. Additionally, a maximum weight clique has the highest sum of weights of its associated vertices, compared to all other cliques that can be found in the given graph. The goal of the MWCP is to discover one of possibly several such maximum weight cliques. The challenge is to efficiently determine which vertices should be included in the clique in order to achieve the maximum weight while maintaining the definition of the clique.

The MWCP is an NP hard problem, indicating that as the weighted graph size grows, the computational effort required to find an optimal solution increases exponentially. Several exact algorithms have been developed to address the MWCP^8–11^ and related problems^12^ so far. These algorithms guarantee optimal solutions by employing techniques like branch and bound that allows them to explore efficiently only a subset of possible maximum weight cliques within the graph, and to quickly prune the unpromising branches. To tackle massive graphs, heuristic algorithms have also been developed to provide weighted cliques within a reasonable time, which is sufficient for most practical applications^13–15^.

In this work, we develop a new exact maximum weight clique algorithm MaxCliqueWeight and its variant MaxCliqueDynWeight, a dynamic variant of the algorithm involving dynamically varying upper bounds as first introduced in^16^, which increase efficiency of the clique-finding algorithm. The algorithms are based on a fast branch and bound algorithm for finding a maximum clique in an undirected graph^16^. We test the newly developed algorithms on random weighted graphs of up to 10,000 vertices and on standard benchmark DIMACS graphs, used in different research fields and industries^17^. We show that our newly developed algorithms are up to three orders of magnitude faster than a comparable Cliquer algorithm^8,18^ on random graphs, with the largest speedup achieved by the MaxCliqueDynWeight algorithm on a graph with 100 vertices and an edge density of 0.95; the MaxCliqueWeight algorithm is up to six orders of magnitude faster on DIMACS graphs, with the highest speedup on the san200_0.7_2 graph with 200 nodes and an edge density of 0.7.

## Methods

### Graph notations

Table 1 gives an overview of the variables used in the developed MaxCliqueWeight algorithm and its MaxCliqueDynWeight variant together with their meaning.

**Table 1:** Graph variables and definitions used in the clique algorithms.

Name	Definitions
G	An input graph G represented by a set of vertices R and a set of adjacent vertices Γ(v) for each vertex v ∈ R
R	A set of input graph vertices represented by numbers 0…n
Γ(v)	A set of vertices adjacent (connected by an edge) to each vertex v ∈ R in the input graph G
Δ(R)	Maximum degree of any vertex in R
C	A set of color classes C[k], k = 1…|R|+ 1, where each contains non-adjacent vertices only; k represents the color of all vertices in the k-th color class
Q	A global variable, a set of graph vertices that represent a (weighted) clique being constructed
W	A set of weights for graph vertices of the input graph (e.g., W[R[i]] denotes the weight of vertex with index i in R)
Qmax	A global variable, a set of graph vertices that represent a maximum weight clique

### A new algorithm to find a maximum weight clique in a weighted undirected graph

We describe a new algorithm MaxCliqueWeight, in which we have extended the basic algorithm, referred to as MaxClique, developed in^16^. The new MaxCliqueWeight algorithm works on vertex-weighted graphs, that is, graphs where each vertex is assigned a weight represented by a positive integer number. The new algorithm finds a maximum weight clique, i.e., a clique with the highest total sum of its weights, in an undirected vertex-weighted graph, as shown in Table 2.

**Table 2:** The initialization steps of the new algorithms MaxCliqueWeight and MaxCliqueDynWeight.

Line	Pseudocode	Description
1	SortDecDeg (R)	Sort vertices in R by decreasing degrees (from the highest to the lowest degree)
2	ColorSortWeight (R)	Set initial colors and wcolors of the input vertices in set R. These are the upper bounds to the size and the weight of a maximum clique, respectively
3	Expand (R)	Start the recursive branch-and-bound tree search for (weight) cliques

The algorithm recursively explores the search tree of the possible weighted cliques, using a branch-and-bound technique to efficiently prune parts of the search space that cannot contain a maximum weight clique. It consists of the new ColorSortWeight procedure (see Table 3) that provides the upper bound to the weight of the clique that can be found at each step of the search tree, and of the Expand procedure (see Table 4), the recursive procedure that performs the branch-and-bound search.

**Table 3:**
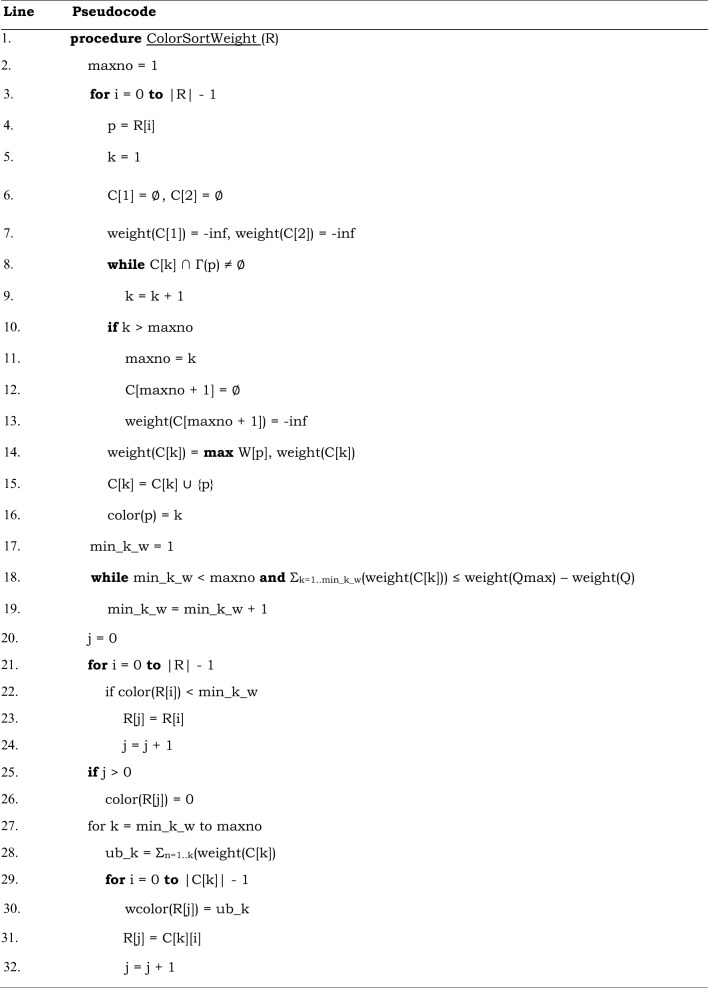
A new approximate coloring algorithm for weighted undirected graphs used in our new maximum weight clique algorithm MaxCliqueWeight and its variant MaxCliqueDynWeight. Global variables are: Q – a set that contains the currently growing weight clique, Qmax – a set that contains the highest weight clique currently found. For definitions of all variables see Table 1.

**Table 4:**
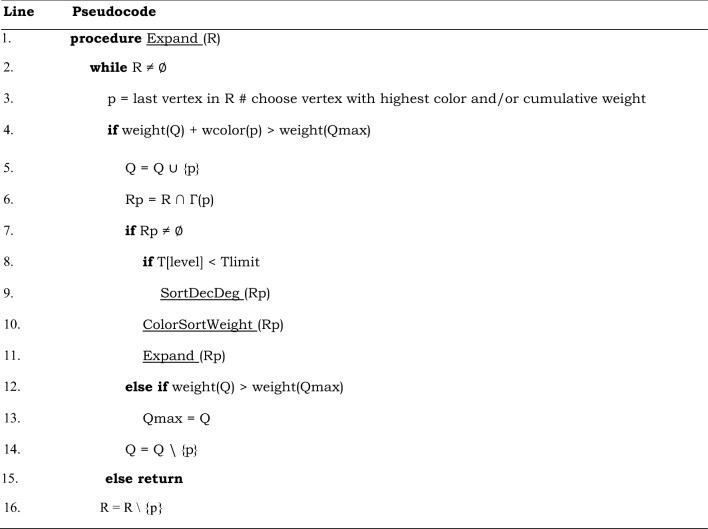
Expand procedure for the new maximum weight clique algorithm MaxCliqueWeight and its variant MaxCliqueDynWeight. Global variables are the set Q that contains the currently growing clique and the set Qmax which contains the largest clique currently found. For definitions of all variables see Table 1.

#### Input

The algorithm takes as input a weighted graph G represented by a set of vertices R, a set of adjacent vertices Γ(v) for each vertex v ∈ R, and a set of weights W, where each vertex v ∈ R has assigned a weight w, which is a positive number (w > 0).

#### Output

The set Qmax consisting of vertices of the maximum weight clique found in the input vertex-weighted graph.

#### Initialization

Vertices in the set R (see Table 2) are sorted by their degrees in decreasing order, so that the first vertex has the highest degree of all vertices in R, and the last one has the lowest degree (see line 1 in Table 2). This order of vertices produces the tightest upper bounds to the size of a maximum weight clique in our experiments (see also^16^). The ColorSortWeight procedure is then called once on the input vertices in the set R (line 2 in Table 2). This procedure efficiently determines the initial upper bound to the weight of a clique (wcolor) for each vertex v∈R if v is selected to form the growing clique.

#### ColorSortWeight procedure: determination of upper bounds to clique weight

The ColorSortWeight procedure takes a set of vertices R as input and partitions these vertices into color classes C, where vertices in the same color class C[k] are not connected by an edge as shown in Table 3. Here, k represents the color of all vertices in color class C[k]. For each vertex p ∈ R, the procedure determines the lowest color k such that no vertex in the k-th color class C[k] is adjacent to p (line 8 in Table 3). If k is greater than the maximum number of colors seen so far represented by the variable maxno (line 10 in Table 3), a new color class is created. Vertex p is then inserted into this color class k and its color k is assigned to it, i.e., color(p) = k. At each step, the weight of color class C[k], initially set to zero, is updated to the weight of the vertex p if the weight of p is larger than the weight of its color class (line 14 in Table 3). This results in each color class C[k] being assigned the maximum weight of its vertices. Thus, any current clique Q consisting of vertices in the set R that is found will have at most k vertices and its weight will be less or equal the sum of the maximum weights of the color classes 1 through k, i.e., weight(Q) ≤ Σ_n=1..k_(weight(C[n])). This condition holds after line 16 in Table 3.

In the next step, the ColorSortWeight procedure determines the color class (min_k_w) below which vertices cannot be used to extend the growing weighted clique based on the difference between the weight of the currently growing clique Q and the weight of the maximum weight clique found so far Qmax (line 18 in Table 3). The value of min_k_w is found iteratively by starting with min_k_w = 1 and increasing it to maxno; the search is stopped when the sum of the weights of the k smallest color classes is greater than the difference between the weight of the maximum clique found so far Qmax and the weight of the currently growing clique Q.

The vertices in the set R with colors less than min_k_w are not going to be used to extend the current clique, therefore they can be kept in the initial ordering. They are copied to the beginning of R in their initial order of decreasing degrees (lines 21–24 in Table 3). Maintaining the initial order of vertices was shown to produce tighter upper bounds than if order is not maintained^19^ and improves efficiency of clique search (see^16^).

On the other hand, vertices in color classes with k greater or equal to min_k_w can form cliques with weights higher than the weight of the maximum weight clique Qmax found so far. These vertices are copied from their respective color classes C, starting from C[min_k_w] and ending with C[maxno], back to the set R in the order in which they appear in each color class (lines 27–32 in Table 3). Each such vertex with color of k is assigned a cumulative weight (wcolor), which is the sum of maximum weights of all color classes 1 to k (see the calculation of ub_k on line 28 in Table 3). A wcolor represents the upper bound to the weight of the clique that can be found in the Expand procedure; wcolor is used for prunning the search tree, which is described next.

#### Expand procedure: finding a maximum weight clique

After the upper bounds of vertices in R are set, the Expand procedure is called. This call is done once during the Initialization phase (see Table 2) as well as at each step during the recursive branch-and bound tree search (line 11 in Table 4) as explained in the following. The Expand procedure explores the search tree of possible weighted cliques, starting with the initially empty set Q representing a set of vertices of the currently growing weighted clique. At each step, Expand selects the vertex p ∈ R with the highest cumulative weight (wcolor) (line 3 in Table 4), which is the last vertex in set R, and removes this vertex p from the set R. If the weight of Q, which is the sum of the weights of all its constituting vertices plus the weight of the selected vertex p is greater than the weight of Qmax, the vertex p is added to Q (line 4 in Table 4).

The subset of vertices Rp ⊂ R, in which each vertex is adjacent to p, is determined (line 6 in Table 4), and if this set Rp is not empty, the ColorSortWeight procedure is called with Rp as an argument. This sets the upper bounds (wcolors) for vertices in set Rp. The Expand procedure is then called recursively with Rp as argument. The recursive calls continue until Rp is empty. If Rp is empty, and the weight of Q is greater than the weight of Qmax (line 12 in Table 4), Qmax is updated to be Q. In any case, if the weight of the candidate clique is greater than Qmax or not, the Expand backtracks removing the added vertex from Q to allow the search along a different branch of the search tree. The result of the Expand procedure is a set Qmax containing vertices of a maximum weight clique that was found in the input vertex-weighted graph.

#### Dynamically varying bounds for greater efficiency

In the MaxCliqueWeight algorithm the calculation of the degrees and sorting of vertices is performed only once with the initial set of vertices R (see line 1 in Table 2). In^16^, we developed a new technique of varying upper bounds that recalculates the degrees of vertices in Rp in the graph induced by these vertices, i.e., G(Rp), at heuristically determined steps of the Expand procedure. Vertices in Rp are then sorted in a decreasing order with respect to their degrees in G(Rp). The graph coloring algorithm thus considers vertices in Rp sorted by their degrees in the induced graph G(Rp) rather than in G, which increases their tightness. Varying upper bounds enables to reduce the number of steps required to find the maximum clique and improve the run time of the algorithm by as much as an order of magnitude on dense graphs, while preserving its superior performance on sparse graphs^16^.

However, the calculation of degrees is computationally expensive (O(|Rp|^2^), therefore we need to determine at which steps this should be performed to decrease the overall running time of the maximum clique search. The heuristic by which we determine the steps where the recalculation of degrees in G(Rp) and resorting of vertices is performed assumes that the calculation time is improved only when the candidate set Rp is large. Obviously, set Rp is larger on initial (lower) levels of the recursion of the Expand procedure than on the higher levels. With the recursion level we denote the number of recursive calls of the Expand procedure from the first call to the current branch. For large candidate sets the computational expense related to the computation of tighter bound is much smaller than the cost of investigating false solutions, which arise when applying less tight bounds.

Therefore, we count the number of steps up to and including each level of the recursion in the Expand procedure and also the number of all steps completed so far. Using these two values, we calculate T[level], which is the fraction of steps up to the current level among all the steps completed so far (see line 8 in Table 4). With a new heuristic parameter, Tlimit, we can then limit the use of tighter bounds (recalculation of degrees) to certain levels. While T[level] is less than Tlimit, we perform the calculations of the degrees and sorting and in the ColorSort algorithm we consider vertices in Rp sorted by their degrees in G(Rp). The Tlimit parameter is set to 0.025 by default^16^, which limits the calculation of degrees to the lower levels of the recursion where Rp is the largest.

### Maximum weight clique search on an example graph

**Figure 1:**
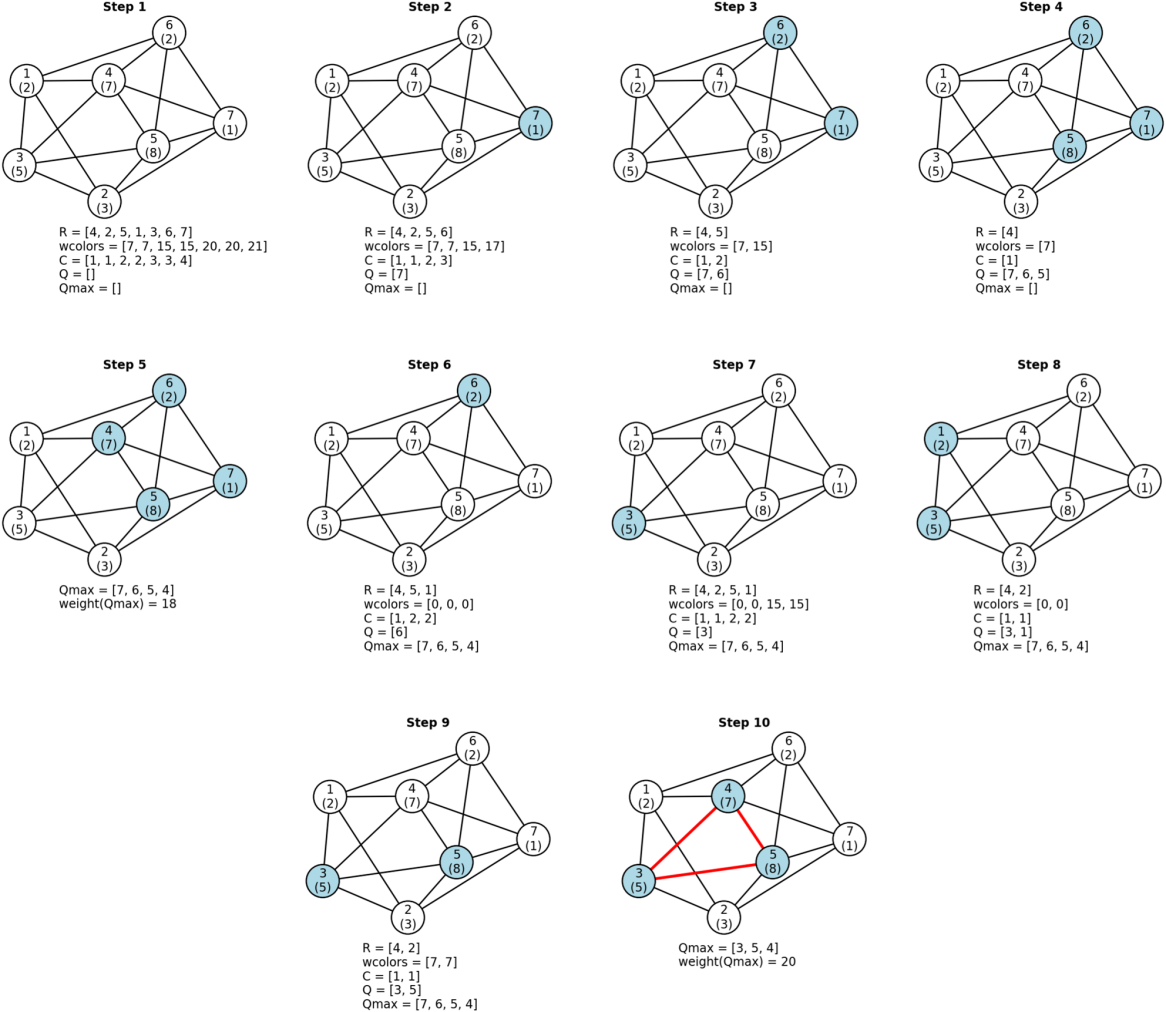
Example calculation with the MaxCliqueWeight algorithm on a weighted graph. Each vertex is represented as a circle and has a vertex number and below it is in parentheses the vertex’s weight. Vertices belonging to the current clique Q are colored in blue. Maximum weight clique is shown with red thick edges. In each step, below the graph are the current values of important variables as they are sampled at the beginning of the Expand procedure (before line 2 in Table 4) for steps 1–4 and 6–9 or at the update of Qmax in the Expand procedure (after line 13 in Table 4) for steps 5 and 10: R—graph vertices that remain to be explored; wcolors—upper bounds to the weight of a clique, where each wcolor corresponds to the vertex in R at the same index in the list; C—color classes determined in the ColorSortWeight procedure, where each color class corresponds to the vertex in R at the same index in the list; Q—vertices of the current growing clique; Qmax—vertices of the current maximum weight clique.

To introduce the novel MaxCliqueWeight algorithm, we illustrate its operation through a step-by-step walkthrough on a sample weighted graph. This graph comprises seven vertices, labeled one through seven and depicted as circles. Each vertex is associated with a positive integer weight, denoted within parentheses, as shown in Figure 1.

In Step 1, we initiate the Expand procedure for the first time (see line 3 in Table 2). At this point, the set R contains vertices of the input graph, sorted by their degrees in descending order. The first vertex in R (vertex no. 4) has five degrees (number of connected edges), while all other vertices have four degrees. The set wcolors, representing the upper bounds for clique weights in the input graph, has been initialized with the ColorSortWeight procedure (as seen in Table 3). Additionally, sets Q and Qmax are empty at this stage.

In Step 2, which marks the next level of the recursion of the Expand procedure, the last vertex in R (vertex no. 7, colored blue in Figure 1) is selected. The condition on line 4 in Table 4, known as the weight bound condition, is satisfied. This condition checks if the upper bound (wcolor) of vertex no. 7, which is wcolor(7) = 21, plus the weight of Q (0) is greater than the weight of Qmax (0). In this case, this condition is met, and vertex no. 7 is added to the set Q, representing the growing clique. Simultaneously, the set R is reduced to contain only the vertices adjacent to vertex no. 7.

In Step 3 and Step 4, the last vertices in R, vertex no. 6 and vertex no. 5, respectively, are added to the set Q. In Step 5, vertex no. 4 is added. Since Rp is empty, indicating that the clique in Q cannot expand further, the set Q is copied into Qmax.

Moving to Step 6, the Expand branch-and-bound procedure backtracks to the recursion level of Step 1. It then begins constructing a new clique Q by selecting the next-to-last vertex in R (vertex no. 6). The weight bound condition on line 4 in Table 4 is satisfied, as the wcolor of vertex no. 6, which is 20, plus the weight of Q (0) is greater than the weight of Qmax (18). Therefore, vertex no. 6 is added to the set Q. However, all the vertices in the current set R (vertices no. 4, 5, and 1) have wcolors equal to zero. Consequently, none of these vertices can extend Q's weight beyond that of Qmax, i.e., the weight of Q (2) plus the wcolor of vertex no. 4, 5, or 1 (0) is less than the weight of Qmax (18).

In Step 7, the Expand procedure backtracks to the recursion level of Step 1, and vertex no. 3 is added to the set Q.

In Step 8 vertex no. 1 is added to the set Q, as the weight of Q (5), which now includes vertex no. 3, plus the wcolor of vertex no. 1 (15), exceeds the weight of Qmax (18). Vertex no. 2 is then considered for addition but is excluded since the weight of Q (7), including vertices no. 3 and 1, plus the wcolor of vertex no. 2 (0), is less than the weight of Qmax (18). Consequently, the algorithm backtracks to the recursion level of Step 7 and attempts to add another vertex.

In Step 9, vertex no. 5 is added to the set Q because the weight bound condition is met. The weight of the set Q (5) plus the wcolor of vertex no. 5 (15) is greater than the weight of Qmax (18). The algorithm then attempts to extend the clique in Q with the next-to-last vertex in R, vertex no. 2, but this fails. This time, the reason for not adding the vertex is the condition on line 12 in Table 4. This condition checks if weight(Q) > weight(Qmax), which is not met as weight(Q) = 13 < weight(Qmax) = 18.

Finally, in Step 10, vertex no. 4 is added to the set Q, as the weight of Q (13) plus the wcolor of vertex no. 4 (7) is greater than the weight of Qmax (18). The weight of the new clique in Q is now 20. Consequently, Qmax is updated to be Q, and Qmax now holds the maximum weight clique (marked red in Figure 1), which is the result of this example calculation.

## Results and discussion

To evaluate the developed maximum weight clique algorithm MaxCliqueWeight and its dynamic variant MaxCliqueDynWeight we have tested them on the test set of random weighted graphs as well as on the test set of DIMACS graphs. We have compared our algorithms to the Cliquer algorithm^8,18^ for finding a maximum weight clique, which is widely used and well established in the research community. We did not investigate some graph size and edge density combinations and did not consider certain DIMACS graphs due to the expected long computation times.

### Generation of weighted graphs for testing

#### Random weighted graphs

The first test set consists of 35 random weighted graphs with 100, 200, 300, 500, 700, 1000, 5000, and 10000 vertices, where we refer to the number of vertices as the size of a graph. Edge densities within each graph size category take discrete values of 0.1, 0.3, 0.5, 0.7, 0.8, 0.9, 0.95, 0.99, where edge density of a graph equals to the probability p of an edge between two graph vertices. For each graph of a given size and for each pair of vertices vi and vj within this graph, if a uniform continuous distribution u(vi, vj) for random numbers on the range [0, 1), with equal probability throughout the range, was less than a probability p, then we connected the vertices vi and vj by an edge.

To each vertex v we assigned a weight, which is a positive integer number. The weight assignment is done as follows. For each vertex v we generated a real value random number according to the normal random number distribution defined as: f(x;μ,σ) = 1/(σ·sqrt(2π))e^(− 1/2·((x − μ)/σ)^2), where μ is the mean and σ is the standard deviation; in our experiments, we set μ to 1,000,000 and σ to 200,000. We checked that the resulting random numbers were positive, i.e., > 0. We then rounded the resulting values to the nearest integer values. In this way, we obtained a random positive integer number, a weight, for each vertex v in a graph.

#### Benchmark graphs from the DIMACS challenge

The second test set consists of 57 graphs from the second DIMACS implementation challenge available at http://dimacs.rutgers.edu^17^. The DIMACS implementation challenges help understand and improve the practical performance of algorithms for important problems, particularly those that are hard in the theoretical sense. It is a publicly available collection of benchmark graphs for testing algorithms, such as the maximum clique, graph coloring, and satisfiability algorithms, which are all NP hard problems. Since the DIMACS graphs are unweighted graphs, we assigned vertex weights to them using the same procedure as described for random graphs in the second paragraph of section for generation of random weighted graphs.

### Random weighted graphs

The results for random weighted graphs are in Table 5. The best performing algorithm is the MaxCliqueDynWeight algorithm, which achieves up to three orders of magnitude speedup compared to the Cliquer algorithm^8,18^ on difficult to solve graph instances characterized by their high edge densities. For example, see random graph with 100 vertices and edge density of 0.95, where MaxCliqueDynWeight achieves 6100x speedup. As the edge density approaches 1.0, our algorithm takes less time, e.g., see graphs with 100 and 200 vertices and edge densities of 0.9 and 0.99 in Table 5. This can be explained by the fact that the closer we get to the density of 1.0, the easier the maximum clique problem becomes, since a graph with an edge density of 1.0 is by definition a maximum clique.

**Table 5:** Calculation times on random weighted graphs for the MaxCliqueWeight and MaxCliqueDynWeight algorithms compared to the Cliquer algorithm. The fastest calculation times and best speedups in each row are in bold. Calculation times are averaged over 100 runs, where each run was performed with randomly shuffled graph vertices as input. Calculation times that were < 1 ms were set to 1 ms, while those exceeding 2 h were set to 2 h.

Graph		MaxCliqueWeight		MaxCliqueDynWeight		Cliquer
Size	Density	Time ± SD [s]	Speedup*	Time ± SD [s]	Speedup*	Time ± SD [s]
100	0.1	0.001 ± 0	1	0.001 ± 0	1	0.001 ± 0
100	0.3	0.001 ± 0	1	0.001 ± 0	1	0.001 ± 0
100	0.5	0.001 ± 0	1	0.001 ± 0	1	0.001 ± 0
100	0.7	0.0041 ± 0.00078	2.2	**0.0028 ± 0.00058**	**3.2**	0.009 ± 0.0013
100	0.8	0.015 ± 0.0025	6.3	**0.01 ± 0.0017**	**9.3**	0.096 ± 0.015
100	0.9	0.053 ± 0.018	46	**0.025 ± 0.006**	**99**	2.4 ± 0.12
100	0.95	0.012 ± 0.0048	4000	**0.0077 ± 0.0025**	**6100**	47 ± 1.1
100	0.99	**0.0019 ± 0.0011**	**770**	0.0024 ± 0.00094	630	1.5 ± 0.08
200	0.1	0.001 ± 0	1	0.001 ± 0	1	0.001 ± 0
200	0.3	0.001 ± 0.0001	1	0.001 ± 0	1	0.001 ± 0.00017
200	0.5	0.016 ± 0.0018	1	**0.013 ± 0.0019**	**1.3**	0.017 ± 0.0015
200	0.7	0.64 ± 0.043	2.2	**0.38 ± 0.033**	**3.7**	1.4 ± 0.09
200	0.8	11 ± 0.67	5.7	**4.9 ± 0.23**	**13**	63 ± 1.7
200	0.9	310 ± 65	> 23	**62 ± 6.2**	** > 120**	> 2 h
200	0.95	2500 ± 710	> 2.9	**300 ± 34**	** > 24**	> 2 h
200	0.99	18 ± 21	> 390	**8.1 ± 2.9**	** > 890**	> 2 h
300	0.1	0.001 ± 0	1	0.001 ± 0	1	0.001 ± 0
300	0.3	**0.0039 ± 0.0006**	**1.2**	0.0041 ± 0.00035	1.1	0.0046 ± 0.00071
300	0.5	0.1 ± 0.011	1.1	**0.085 ± 0.01**	**1.3**	0.11 ± 0.01
300	0.7	14 ± 0.87	2.4	**7.8 ± 0.41**	**4.3**	33 ± 0.85
300	0.8	1700 ± 74	> 4.2	**480 ± 10**	** > 15**	> 2 h
300	0.99	5500 ± 1000	> 1.3	**3400 ± 1000**	** > 2.1**	> 2 h
500	0.1	0.001 ± 0	1.6	**0.001 ± 0**	**1.6**	0.0016 ± 0.0005
500	0.3	0.031 ± 0.004	0.88	0.03 ± 0.003	0.93	**0.028 ± 0.0029**
500	0.5	2.6 ± 0.11	1.1	**1.9 ± 0.1**	**1.5**	2.9 ± 0.12
500	0.7	2200 ± 110	2.8	**930 ± 41**	**6.6**	6100 ± 160
700	0.1	0.0024 ± 0.00074	1.8	**0.0022 ± 0.00057**	**1.9**	0.0043 ± 0.001
700	0.3	0.13 ± 0.015	0.94	0.13 ± 0.016	0.97	**0.12 ± 0.014**
700	0.5	22 ± 0.83	0.94	**16 ± 0.58**	**1.3**	21 ± 0.52
1000	0.1	**0.0065 ± 0.0011**	**1.5**	0.0066 ± 0.0014	1.5	0.0099 ± 0.002
1000	0.3	0.64 ± 0.035	0.77	0.63 ± 0.062	0.79	**0.5 ± 0.042**
1000	0.5	250 ± 4.4	1	**180 ± 3.6**	**1.4**	250 ± 6.8
5000	0.1	1 ± 0.091	0.8	1.3 ± 0.082	0.63	**0.81 ± 0.079**
5000	0.3	3300 ± 71	0.66	3000 ± 76	0.73	**2200 ± 55**
10,000	0.1	14 ± 0.32	0.65	15 ± 0.32	0.59	**9.1 ± 0.34**

*Speedups are calculated by dividing the Cliquer algorithm’s calculation time with the MaxCliqueWeight’s or MaxCliqueDynWeight’s calculation time.

Generally, our MaxCliqueWeight and MaxCliqueDynWeight algorithms perform best on dense graphs with edge densitites 0.5–0.99, while they also still retain good performance on sparser graphs. The large speedup of our algorithms is most likely due to the use of efficient upper bound computation based on graph coloring, which allows us to efficiently prune large parts of the search tree compared to the Cliquer algorithm, which has no such upper bounds.

### DIMACS benchmark graphs

Next, we tested the developed maximum weight clique algorithms on DIMACS graphs, used in standard benchmarking of maximum clique algorithms. We added random vertex weights to DIMACS graphs as described. The results are in Table 6.

**Table 6:** Calculation times on weighted DIMACS graphs for the MaxCliqueWeight and MaxCliqueDynWeight algorithms compared to the Cliquer algorithm. The fastest calculation times and best speedups in each row are in bold. Calculation times are averaged over 100 runs, each run performed with randomly shuffled graph vertices. Calculation times that were < 1 ms were set to 1 ms, while those exceeding 2 h were set to 2 h.

Graph			MaxCliqueWeight		MaxCliqueDynWeight		Cliquer
Name	Size	Density	Time ± SD [s]	Speedup*	Time ± SD [s]	Speedup*	Time ± SD [s]
C125-9	125	0.9	0.72 ± 0.18	180	**0.32 ± 0.059**	**410**	130 ± 3.7
C250-9	250	0.9	> 2 h	1	**4500 ± 240**	** > 1.6**	> 2 h
MANN_a27	378	0.99	**1000 ± 1600**	** > 6.9**	2400 ± 2100	> 3	> 2 h
MANN_a9	45	0.93	0.001 ± 0	9.3	**0.001 ± 0**	**9.3**	0.0094 ± 0.0026
brock200_1	200	0.75	1.5 ± 0.18	3.3	**0.78 ± 0.11**	**6.3**	4.9 ± 0.2
brock200_2	200	0.5	0.014 ± 0.0014	1	**0.012 ± 0.00095**	**1.2**	0.014 ± 0.0014
brock200_3	200	0.61	0.084 ± 0.009	1.5	**0.065 ± 0.0066**	**1.9**	0.13 ± 0.012
brock200_4	200	0.66	0.22 ± 0.023	1.2	**0.15 ± 0.018**	**1.8**	0.27 ± 0.024
brock400_1	400	0.75	1500 ± 190	4.7	**570 ± 75**	**12**	6900 ± 160
brock400_2	400	0.75	1500 ± 220	3.1	**580 ± 87**	**7.8**	4500 ± 130
brock400_3	400	0.75	760 ± 290	3.8	**290 ± 110**	**10**	2900 ± 72
brock400_4	400	0.75	550 ± 220	1.3	**230 ± 92**	**3**	690 ± 15
brock800_3	800	0.65	> 2 h	1	**5800 ± 670**	** > 1.2**	> 2 h
brock800_4	800	0.65	7100 ± 100	1	**4500 ± 840**	** > 1.6**	> 2 h
c-fat200-1	200	0.08	0.001 ± 0	1	0.001 ± 0	1	0.001 ± 0
c-fat200-2	200	0.16	0.001 ± 0	1	0.001 ± 0	1	0.001 ± 0
c-fat200-5	200	0.43	0.026 ± 0.015	0.04	0.001 ± 0	1	0.001 ± 0
c-fat500-1	500	0.04	0.001 ± 0	1	0.001 ± 0	1	0.001 ± 0
c-fat500-10	500	0.37	0.011 ± 0.0081	0.37	**0.0039 ± 0.0012**	**1**	0.004 ± 0.001
c-fat500-2	500	0.07	0.001 ± 0	1	0.001 ± 0	1	0.001 ± 0.00014
c-fat500-5	500	0.19	0.0018 ± 0.0014	0.84	**0.0011 ± 0.0003**	**1.4**	0.0015 ± 0.0005
gen200-p0-9–44	200	0.9	47 ± 17	> 150	**19 ± 5.9**	** > 380**	> 2 h
gen200-p0-9–55	200	0.9	13 ± 7.1	420	**3 ± 1.4**	**1800**	5300 ± 130
hamming6-2	64	0.9	0.001 ± 0	1	0.001 ± 0	1	0.001 ± 0
hamming6-4	64	0.35	0.001 ± 0	1	0.001 ± 0	1	0.001 ± 0
hamming8-2	256	0.97	9.9 ± 8.1	690	**2.2 ± 0.8**	**3000**	6800 ± 180
hamming8-4	256	0.64	0.046 ± 0.006	6	**0.043 ± 0.005**	**6.4**	0.27 ± 0.022
johnson16-2–4	120	0.76	**0.016 ± 0.0086**	**15**	0.046 ± 0.0082	5.2	0.24 ± 0.024
johnson8-2–4	28	0.56	0.001 ± 0	1	0.001 ± 0	1	0.001 ± 0
johnson8-4–4	70	0.77	0.001 ± 0	1	0.001 ± 0	1	0.001 ± 0.0001
keller4	171	0.65	0.018 ± 0.0036	7.8	**0.011 ± 0.001**	**12**	0.14 ± 0.013
p_hat1000-1	1000	0.24	0.38 ± 0.028	1.1	**0.34 ± 0.036**	**1.2**	0.41 ± 0.024
p_hat1000-2	1000	0.49	> 2 h	1	**1800 ± 150**	** > 4.1**	> 2 h
p_hat1500-1	1500	0.25	4.2 ± 0.13	0.79	3.5 ± 0.099	0.94	**3.3 ± 0.12**
p_hat300-1	300	0.24	0.0022 ± 0.00039	1.4	**0.0021 ± 0.00034**	**1.4**	0.003 ± 0.00028
p_hat300-2	300	0.49	0.1 ± 0.016	5.4	**0.059 ± 0.0077**	**9.3**	0.55 ± 0.047
p_hat300-3	300	0.74	25 ± 2.7	37	**6.4 ± 0.63**	**150**	940 ± 24
p_hat500-1	500	0.25	0.019 ± 0.0025	1.2	**0.018 ± 0.0018**	**1.3**	0.023 ± 0.0023
p_hat500-2	500	0.5	9.5 ± 0.83	11	**2.7 ± 0.2**	**37**	100 ± 2.5
p_hat500-3	500	0.75	> 2 h	1	**2300 ± 110**	** > 3.1**	> 2 h
p_hat700-1	700	0.25	0.096 ± 0.011	0.81	0.082 ± 0.0085	0.95	**0.077 ± 0.0078**
p_hat700-2	700	0.5	250 ± 15	> 29	**47 ± 1.7**	** > 150**	> 2 h
san1000	1000	0.5	9.3 ± 0.69	> 780	**0.25 ± 0.018**	** > 29,000**	> 2 h
san200_0.7_1	200	0.7	0.055 ± 0.041	970	**0.0071 ± 0.0039**	**7400**	53 ± 1.2
san200_0.7_2	200	0.7	**0.0056 ± 0.00074**	**1,000,000**	0.0061 ± 0.0005	930,000	5700 ± 190
san200_0.9_1	200	0.9	0.17 ± 0.14	> 42,000	**0.11 ± 0.048**	** > 68,000**	> 2 h
san200_0.9_2	200	0.9	7 ± 9.3	> 1000	**1 ± 0.47**	** > 7200**	> 2 h
san200_0.9_3	200	0.9	82 ± 43	> 88	**20 ± 7.8**	** > 360**	> 2 h
san400_0.5_1	400	0.5	0.027 ± 0.0083	2300	**0.0076 ± 0.0011**	**8200**	62 ± 1.5
san400_0.7_1	400	0.7	15 ± 12	> 490	**0.98 ± 0.45**	** > 7300**	> 2 h
san400_0.7_2	400	0.7	35 ± 24	> 200	**3.3 ± 1.6**	** > 2200**	> 2 h
san400_0.7_3	400	0.7	37 ± 9.6	> 200	**8.9 ± 1.1**	** > 810**	> 2 h
san400_0.9_1	400	0.9	**980 ± 1100**	** > 7.4**	1100 ± 1100	> 6.7	> 2 h
sanr200_0.7	200	0.7	0.52 ± 0.034	3.3	**0.31 ± 0.025**	**5.6**	1.7 ± 0.065
sanr200_0.9	200	0.9	310 ± 47	> 23	**53 ± 4.2**	** > 140**	> 2 h
sanr400_0.5	400	0.5	0.7 ± 0.041	1.2	**0.54 ± 0.024**	**1.5**	0.83 ± 0.036
sanr400_0.7	400	0.7	280 ± 8.5	2.5	**120 ± 2.8**	**5.9**	700 ± 18

*Speedups are calculated by dividing the Cliquer algorithm’s calculation time with the MaxCliqueWeight’s or MaxCliqueDynWeight’s calculation time.

The clear winner among the three algorithms tested is the MaxCliqueDynWeight algorithm. It is especially well suited for denser graphs with edge densities 0.5-0.99, where it achieves speedups of several orders of magnitude compared to Cliquer algorithm. For examples, see »san« and »sanr« graphs in Table 6. On the other hand, for sparse graphs with edge densities 0.1-0.3, which are easier to solve, characterized by the calculation times typically under a second, our algorithm still achieves comparable speeds to the Cliquer algorithm.

### Effect of ordering of graph vertices on algorithms’ performances

To test the effect of the order of input graph vertices on algorithms’ performances, every calculation on random and on DIMACS weighted graphs was repeated 100 times. Each time the input graph vertices were randomly shuffled. This was done before the initialization steps shown in Table 2. The shuffling only changed the position of vertices (vertex numbers) in each input graph, and not the structure of each graph.

We observe that for larger and denser graphs, the effect of initial order on calculation time can be large, e.g., see standard deviations for random graph with 300 vertices and edge density of 0.99 in Table 5, or the MANN_a27 graph with 378 vertices and edge density of 0.99 in Table 6. In these cases, measuring the algorithms’ speed with only one ordering of graph vertices could easily result in wrong assesment of this algorithm’s performance. If we tested with one particular graph vertices ordering for MANN_a27 graph, we could wrongly assign MaxCliqueDynWeight algorithm as the fastest algorithm, while the averaged calculation time shows that MaxCliqueWeight is the winner in this case.

For the graphs MANN_a27, brock800_3, brock800_4, san400_0.9_1 (see Table 6) and the random graph with a size of 300 and an edge density of 0.99 (see Table 5), the high standard deviations remain high even if the number of repetitions is increased from ten to one hundred. This indicates that the high standard deviations in these cases are most likely due to the dependence of our algorithms on the initial order of the vertices and not due to a low number of repetitions.

Shuffling graph vertices and multiple repetitions are not common when testing clique algorithms, but can significantly affect the measurements. Shuffling of graph vertices has already been proposed for this reason^20^. Therefore, we recommend testing all maximum weight clique algorithms and other graph algorithms with different initial orders of graph vertices to assess the effects of the different ordering on computational efficiency.

To test whether these results are representative for graphs of the given size and density or whether they are due to idiosyncratic properties of the graphs, we performed additional experiments for random and DIMACS graphs. For each random graph size and density, we generated 100 new random graphs so that we randomized the edges and weights. For each DIMACS graph, we only applied the generation of random weights 100 times to each graph type, since we could not randomize the edges of these graphs as this would destroy their inherent structures. The average calculation times of the tested algorithms for random and DIMACS weighted graphs, respectively, can be found in Supplementary Tables S1 and S2.

The calculation times and speedups obtained with this new method follow a very similar trend to those we observed with random vertex shuffling, which can be seen by comparing Table 5 with Supplementary Table S1 and Table 6 with Supplementary Table S2. The only exception is the san400_0.9_1 graph, which is solved much faster by our algorithm when randomizing vertex order than when randomizing vertex weights. We have previously observed that the initial order of vertices has a particular impact on the performance of maximum clique algorithms for this particular graph^16^.

## Conclusions

In this work, we describe a new maximum weight clique algorithm MaxCliqueWeight and its variant MaxCliqueDynWeight algorithm. The MaxCliqueDynWeight algorithm proves to be the faster of the two algorithms for most random and DIMACS weighted graphs, and is significantly faster than the widely used Cliquer algorithm for dense graphs. Our algorithm is particularly well suited for dense graphs with edge densities 0.5-0.99, but it retains most of its speed for sparser graphs as well. The developed algorithm finds diverse applications across various domains, such as drug discovery and bioinformatics. It holds the potential to significantly accelerate the development of novel drugs through computer-based algorithms.

### Supplementary Information

Supplementary Information.

## Data Availability

All relevant data are available at http://insilab.org/maxcliqueweight.
