# Supplementary material for: An exact algorithm to find a maximum weight clique in a weighted undirected graph
Source: Sci Rep. 2024 Apr 20;14:9118. doi: 10.1038/s41598-024-59689-x (PMC11032405; doi:10.1038/s41598-024-59689-x)
Supplement: Supplementary file 1 — Supplementary Information. [file 41598_2024_59689_MOESM1_ESM.pdf]

Supplementary Information

An exact algorithm to find a maximum weight clique in a weighted undirected graph

Kati Rozman[1], An Ghysels[2], Dušanka Janežič[1,*] and Janez Konc[1,3,4,*]

[1]*University of Primorska, Faculty of Mathematics, Natural Sciences and Information Technologies, Glagoljaška ulica 8, SI-6000 Koper, Slovenia*

[2]*Ghent University, IBiTech – BioMMedA group, Corneel Heymanslaan 10, entrance 36, 9000 Gent, Belgium*

[3]*National Institute of Chemistry, Theory Department, Hajdrihova 19, SI-1000 Ljubljana, Slovenia*

[4]*University of Ljubljana, Faculty of Pharmacy, Aškerčeva 7, SI-1000 Ljubljana, Slovenia*

*E-mail: konc@cmm.ki.si, dusanka.janezic@upr.si

Table S1. Calculation times on random weighted graphs for the MaxCliqueWeight and MaxCliqueDynWeight algorithms compared to the Cliquer algorithm. The fastest calculation times and best speedups in each row are in bold. Calculation times are averaged over 100 runs, where in each run a new random graph was generated by randomizing its edges and weights. Calculation times that were <1 ms were set to 1 ms, while those exceeding 2 hours were set to 2 hours.

Graph		MaxCliqueWeight		MaxCliqueDynWeight		Cliquer
Size	Density	Time ± SD [s]	Speedup*	Time ± SD [s]	Speedup*	Time ± SD [s]
100	0.1	0.001 ± 0	1	0.001 ± 0	1	0.001 ± 0
100	0.3	0.001 ± 0	1	0.001 ± 0	1	0.001 ± 0
100	0.5	0.001 ± 0	1	0.001 ± 0	1	0.001 ± 0
100	0.7	0.0035 ± 0.0012	3	**0.0026 ± 0.00081**	**4**	0.01 ± 0.0032
100	0.8	0.016 ± 0.006	5.8	**0.01 ± 0.0034**	**9.2**	0.094 ± 0.037
100	0.9	0.056 ± 0.029	57	**0.023 ± 0.0094**	**140**	3.2 ± 1.5
100	0.95	0.034 ± 0.031	1600	**0.016 ± 0.0098**	**3400**	56 ± 53
100	0.99	0.0018 ± 0.0026	52000	**0.0017 ± 0.0014**	**57000**	96 ± 550
200	0.1	0.001 ± 0	1	0.001 ± 0	1	0.001 ± 0
200	0.3	0.001 ± 0	1	0.001 ± 0	1	0.001 ± 0.0001
200	0.5	0.013 ± 0.002	1.2	**0.011 ± 0.0015**	**1.5**	0.017 ± 0.0031
200	0.7	0.58 ± 0.11	2.5	**0.34 ± 0.064**	**4.2**	1.4 ± 0.31
200	0.8	11 ± 2.6	7	**4.2 ± 0.82**	**18**	74 ± 21
200	0.9	720 ± 310	>10	**120 ± 45**	**>60**	>2 h
200	0.95	2700 ± 1600	>2.6	**300 ± 180**	**>24**	>2 h
200	0.99	4.1 ± 11	>1700	**2.2 ± 3.8**	**>3300**	>2 h
300	0.1	0.001 ± 0	1	0.001 ± 0	1	0.001 ± 0
300	0.3	**0.0038 ± 0.00074**	**1.1**	0.0039 ± 0.00069	1.1	0.0042 ± 0.0008
300	0.5	0.12 ± 0.017	1.2	**0.1 ± 0.012**	**1.4**	0.14 ± 0.022
300	0.7	18 ± 2.9	2.6	**9.4 ± 1.3**	**5**	47 ± 8.3
300	0.8	1200 ± 260	5.2	**360 ± 59**	**17**	6100 ± 980
300	0.99	3100 ± 2000	>2.3	**2600 ± 1800**	**>2.8**	>2 h
500	0.1	0.001 ± 0	1.6	**0.001 ± 0**	**1.6**	0.0016 ± 0.00053
500	0.3	0.03 ± 0.0037	0.97	0.03 ± 0.0042	0.98	**0.029 ± 0.0038**
500	0.5	2.5 ± 0.23	1.1	**1.8 ± 0.14**	**1.6**	2.8 ± 0.26
500	0.7	2400 ± 270	2.5	**1000 ± 120**	**5.8**	5900 ± 770
700	0.1	0.0022 ± 0.00053	1.8	**0.0022 ± 0.00055**	**1.9**	0.0041 ± 0.001
700	0.3	0.13 ± 0.018	0.89	0.13 ± 0.016	0.94	**0.12 ± 0.017**
700	0.5	22 ± 1.7	1.1	**16 ± 1.2**	**1.5**	24 ± 2.6
1000	0.1	**0.0066 ± 0.0012**	**1.5**	0.0067 ± 0.0012	1.4	0.0097 ± 0.0018
1000	0.3	0.65 ± 0.06	0.82	0.63 ± 0.068	0.85	**0.53 ± 0.058**
1000	0.5	250 ± 16	1.1	**180 ± 12**	**1.5**	280 ± 25
5000	0.1	1 ± 0.06	0.83	1.3 ± 0.06	0.65	**0.85 ± 0.07**
5000	0.3	3400 ± 96	0.71	3100 ± 93	0.78	**2400 ± 130**
10000	0.1	14 ± 0.56	0.69	16 ± 0.57	0.62	**9.6 ± 0.48**

* Speedups are calculated by dividing the Cliquer algorithm's calculation time with the MaxCliqueWeight's or MaxCliqueDynWeight's calculation time.

Table S2. Calculation times on weighted DIMACS graphs for the MaxCliqueWeight and MaxCliqueDynWeight algorithms compared to the Cliquer algorithm. The fastest calculation times and best speedups in each row are in bold. Calculation times are averaged over 100 runs for each graph, where in each run vertex weights were generated randomly. Calculation times that were <1 ms were set to 1 ms, while those exceeding 2 hours were set to 2 h.

Graph			MaxCliqueWeight		MaxCliqueDynWeight		Cliquer
Name	Size	Density	Time ± SD [s]	Speedup*	Time ± SD [s]	Speedup*	Time ± SD [s]
C125-9	125	0.9	0.5 ± 0.19	120	**0.21 ± 0.078**	**300**	61 ± 25

C250-9	250	0.9	>2 h	1	**3800 ± 770**	**>1.9**	>2 h
MANN_a27	378	0.99	**410 ± 870**	**>18**	2000 ± 1900	>3.5	>2 h
MANN_a9	45	0.93	0.001 ± 0	9.2	**0.001 ± 0**	**9.2**	0.0092 ± 0.0038
brock200_1	200	0.75	1.7 ± 0.25	3.8	**0.91 ± 0.13**	**6.9**	6.3 ± 1.2
brock200_2	200	0.5	0.011 ± 0.0018	1.2	**0.0092 ± 0.0014**	**1.5**	0.014 ± 0.0029
brock200_3	200	0.61	0.067 ± 0.013	1.8	**0.051 ± 0.0096**	**2.3**	0.12 ± 0.022
brock200_4	200	0.66	0.22 ± 0.033	1.8	**0.14 ± 0.022**	**2.7**	0.39 ± 0.081
brock400_1	400	0.75	2000 ± 420	2.9	**750 ± 150**	**7.9**	6000 ± 920
brock400_2	400	0.75	1300 ± 320	3.4	**490 ± 130**	**9.2**	4500 ± 1300
brock400_3	400	0.75	860 ± 180	3	**320 ± 61**	**8.1**	2600 ± 1400
brock400_4	400	0.75	390 ± 110	3.7	**160 ± 43**	**8.7**	1400 ± 860
brock800_2	800	0.65	>2 h	1	**6300 ± 620**	**>1.1**	>2 h
brock800_3	800	0.65	>2 h	1	**6500 ± 480**	**>1.1**	>2 h
brock800_4	800	0.65	6200 ± 930	>1.2	**4800 ± 1000**	**>1.5**	>2 h
c-fat200-1	200	0.08	0.001 ± 0	1	0.001 ± 0	1	0.001 ± 0
c-fat200-2	200	0.16	0.001 ± 0	1	0.001 ± 0	1	0.001 ± 0
c-fat200-5	200	0.43	0.017 ± 0.021	0.06	0.001 ± 0.0001	0.99	0.001 ± 0
c-fat500-1	500	0.04	0.001 ± 0	1	0.001 ± 0	1	0.001 ± 0.0001
c-fat500-10	500	0.37	0.0057 ± 0.002	0.73	**0.0037 ± 0.001**	**1.1**	0.0042 ± 0.0034
c-fat500-2	500	0.07	0.001 ± 0	1	0.001 ± 0	1	0.001 ± 0.00017
c-fat500-5	500	0.19	0.0016 ± 0.00065	1.1	**0.001 ± 0.00022**	**1.7**	0.0018 ± 0.00083
gen200-p0-9-44	200	0.9	50 ± 21	>140	**22 ± 7.6**	**>330**	>2 h
gen200-p0-9-55	200	0.9	4.3 ± 1.5	330	**1.8 ± 0.49**	**800**	1400 ± 1500
hamming6-2	64	0.9	0.001 ± 0	1	0.001 ± 0	1	0.001 ± 0.0001
hamming6-4	64	0.35	0.001 ± 0	1	0.001 ± 0	1	0.001 ± 0
hamming8-2	256	0.97	160 ± 230	24	**2.3 ± 1**	**1700**	3900 ± 1700
hamming8-4	256	0.64	0.094 ± 0.018	2.9	**0.048 ± 0.0078**	**5.8**	0.28 ± 0.05
johnson16-2-4	120	0.76	**0.023 ± 0.0087**	**11**	0.052 ± 0.013	4.7	0.25 ± 0.03
johnson8-2-4	28	0.56	0.001 ± 0	1	0.001 ± 0	1	0.001 ± 0
johnson8-4-4	70	0.77	0.001 ± 0	1	0.001 ± 0	1	0.001 ± 0.0001
keller4	171	0.65	0.017 ± 0.0028	6.9	**0.011 ± 0.0014**	**11**	0.12 ± 0.02
p_hat1000-1	1000	0.24	0.4 ± 0.035	0.91	**0.35 ± 0.036**	**1**	0.37 ± 0.039
p_hat1000-2	1000	0.49	>2 h	>1	**1900 ± 630**	**>3.8**	>2 h
p_hat1500-1	1500	0.25	4.1 ± 0.19	0.84	3.5 ± 0.15	1	3.5 ± 0.34
p_hat300-1	300	0.24	0.0021 ± 0.00034	1.2	**0.0021 ± 0.00029**	**1.2**	0.0026 ± 0.00055
p_hat300-2	300	0.49	0.12 ± 0.038	6.4	**0.065 ± 0.018**	**12**	0.79 ± 0.24
p_hat300-3	300	0.74	31 ± 9.4	45	**8.1 ± 2.1**	**170**	1400 ± 520
p_hat500-1	500	0.25	0.02 ± 0.0021	1	**0.019 ± 0.0019**	**1.1**	0.021 ± 0.0029
p_hat500-2	500	0.5	11 ± 3.2	16	**3.4 ± 0.98**	**51**	170 ± 100
p_hat500-3	500	0.75	>2 h	1	**1500 ± 550**	**>4.8**	>2 h
p_hat700-1	700	0.25	0.089 ± 0.012	0.9	0.08 ± 0.012	1	0.08 ± 0.013
p_hat700-2	700	0.5	310 ± 110	17	**50 ± 13**	**110**	5400 ± 1700
san1000	1000	0.5	7.7 ± 2.3	>930	**0.27 ± 0.072**	**>26000**	>2 h
san200_0.7_1	200	0.7	0.014 ± 0.0056	12000	**0.004 ± 0.0015**	**42000**	170 ± 200
san200_0.7_2	200	0.7	**0.0059 ± 0.0011**	**770000**	0.0064 ± 0.0012	710000	4600 ± 1100
san200_0.9_1	200	0.9	0.067 ± 0.032	>110000	**0.048 ± 0.026**	**>150000**	>2 h
san200_0.9_2	200	0.9	21 ± 7.8	97	**1.9 ± 0.98**	**1000**	2000 ± 1600
san200_0.9_3	200	0.9	210 ± 69	>35	**44 ± 13**	**>160**	>2 h
san400_0.5_1	400	0.5	0.019 ± 0.0064	4200	**0.0059 ± 0.0017**	**14000**	80 ± 28
san400_0.7_1	400	0.7	20 ± 9	>360	**0.64 ± 0.096**	**>11000**	>2 h
san400_0.7_2	400	0.7	34 ± 7.6	>210	**3.2 ± 0.56**	**>2200**	>2 h
san400_0.7_3	400	0.7	43 ± 11	>170	**9.8 ± 1.9**	**>730**	>2 h
san400_0.9_1	400	0.9	>2 h	1	**2500 ± 790**	**>2.9**	>2 h
sanr200_0.7	200	0.7	0.52 ± 0.081	2.4	**0.3 ± 0.043**	**4.1**	1.2 ± 0.25
sanr200_0.9	200	0.9	570 ± 210	>13	**83 ± 25**	**>87**	>2 h
sanr400_0.5	400	0.5	0.65 ± 0.051	1.2	**0.51 ± 0.046**	**1.5**	0.76 ± 0.095

sanr400_0.7	400	0.7	260 ± 28	2.6	**110 ± 12**	**5.9**	670 ± 110

[*] Speedups are calculated by dividing the Cliquer algorithm's calculation time with the MaxCliqueWeight's or MaxCliqueDynWeight's calculation time.
